# Successful treatment of paraneoplastic hypercalcemia in a patient with giant condyloma acuminatum: a case report

**DOI:** 10.1186/1752-1947-7-251

**Published:** 2013-11-07

**Authors:** Thomas Linnemann, Frauke Müller, Mathias Löhnert, Peter Hirnle, Martin Görner

**Affiliations:** 1 Department of Hematology and Oncology, Academic Teaching Hospital Bielefeld, Teutoburger Str. 50, Bielefeld 33604, Germany; 2 Department of Surgery and Coloproctology, Academic Teaching Hospital Bielefeld Rosenhöhe, An der Rosenhöhe 27, Bielefeld 33647, Germany; 3 Department of Radiotherapy, Academic Teaching Hospital Bielefeld, Teutoburger Strasse 50, Bielefeld 33604, Germany

**Keywords:** Paraneoplastic hypercalcemia, Buschke-Löwenstein tumor, Giant condyloma acuminatum

## Abstract

**Introduction:**

While paraneoplastic syndromes in patients with malignant and metastasizing tumors are common, they are rarely associated with skin tumors showing predominantly local growth patterns. This case report relates to a patient with giant condyloma acuminatum, also called Buschke-Löwenstein tumor, with paraneoplastic hypercalcemia, who was successfully treated with conservative treatment.

**Case presentation:**

The patient in question is a 48-year-old German man with a giant periscrotal tumor. Before and during the therapy, two episodes of symptomatic hypercalcemia occurred, which were successfully treated by bisphosphonates, intravenous fluids and diuretics. No evidence of lytic bone affection was found.

**Conclusions:**

Paraneoplastic hypercalcemia may occur in patients who have a Buschke-Löwenstein tumor. For patients, where surgery is not an option, established medical therapies like bisphosphonates may be useful in addition to diuretics and infusions.

## Introduction

Paraneoplastic syndromes occur commonly and are well-known. They may appear before or during tumor manifestation. Different types involving different tissues, for example dermatological, neuronal or endocrinological paraneoplastic syndromes, are known. An incidence up to 10% of paraneoplastic hypercalcemia is reported in patients with lung cancer. It is also commonly seen in aggressive cancers such as breast cancer or multiple myeloma [1]. Paraneoplastic hypercalcemia may appear without evidence of bone metastases [2]. It is the most common metabolism-disorder that is induced by a tumor-associated peptide-releasing hormone [3]. Meyer-Heim *et al*. assumed a paraneoplastic prevalence in 40% of hypercalcemia [4]. Buschke-Löwenstein tumors are rare and usually grow slowly. They are thought to be induced by the human papilloma virus (HPV), most commonly type 6 and 11 [5]. Usually, it is associated with extensive local infiltration. Most authors consider it to be a verrucous carcinoma, which rarely metastasizes [6].

## Case presentation

**Figure 1:**
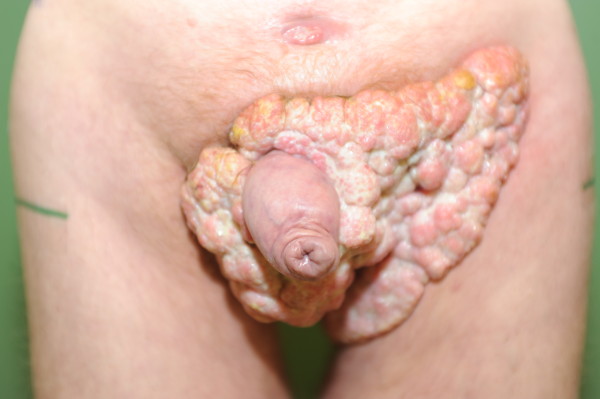
**Tumor before starting therapy**. This is our first picture showing the tumor mass before starting therapy.

**Figure 2:**
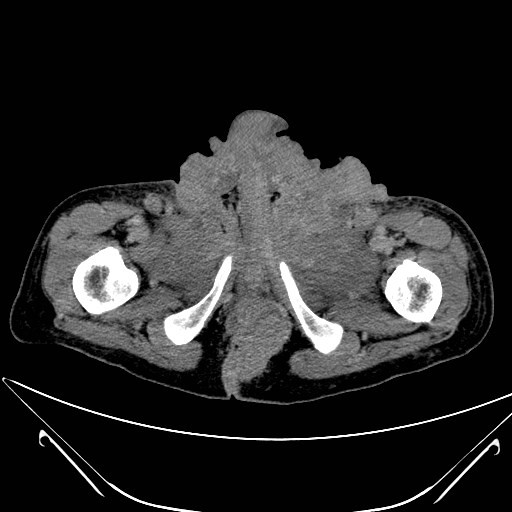
**Extract of pelvis computed tomography scan showing a destructive, polycyclic growing tumor**. This is a picture of a computed tomography scan that we made to exclude bone metastasis. It shows the tumor mass, growing from the anal region (below in the picture) to the groin region (above). Furthermore it shows the local destructive grow-pattern.

A 48-year-old German man was admitted with a large scrotal tumor. He had noticed a tumor mass in his right groin 10 years prevoiusly. After a decade of steady state, the tumor was growing rapidly in both his groins and perineum (Figure 1). Our patient was unable to sit because of pain and tumor mass. After a histological examination, a giant condyloma acuminatum was diagnosed. On examination, the patient was in good condition (Karnofsky-Index: 90%, size: 189cm, weight: 75kg). Further examination showed enlarged groin lymph nodes on both sides and a large cauliflower-like, polycyclic scrotal tumor reaching from pubic eminence to anal fissure (width: 20cm, height: 18cm, depth: 25cm). His medical history reported fatigue, but no neuromuscular weakness, polyuria, polydipsia, nausea, vomiting or psychotic phenomena. Arrhythmia was not observed. A supplement of cholecalciferol was not taken. His laboratory results were unremarkable except for a hypercalcemia (Ca^2+^= 3.64mmol/l). A lower than normal level (LNL) of parathyroid hormone (PTH) and phosphate (parathyroid hormone 9.9 pg/mL, LNL 15 pg/mL; phoshate 2.2mg/dL, LNL 2.7mg/dL) was found (Table 1). His bone scan was normal and a computed tomography scan of his pelvis did not show any evidence for bone affection (Figure 2). Human immunodeficiency virus (HIV) was excluded.

**Table 1:** Results of the patient’s calcium metabolism

**Serum concentration**	**First stay, first results**	**First stay, final results**	**Second stay, first results**	**Second stay, final results**
calcuim (mmol/l)	3.64	2.56	2.91	2.44
phosphate (mg/dl)		2.20	3.10	
parathormon (pg/ml)		9.5		

This table shows the serum calcium levels during the two hospital stays of our patient. Phosphate and parathormon levels were added to show that there was no primary hyperparathyroidism.

**Figure 3:**
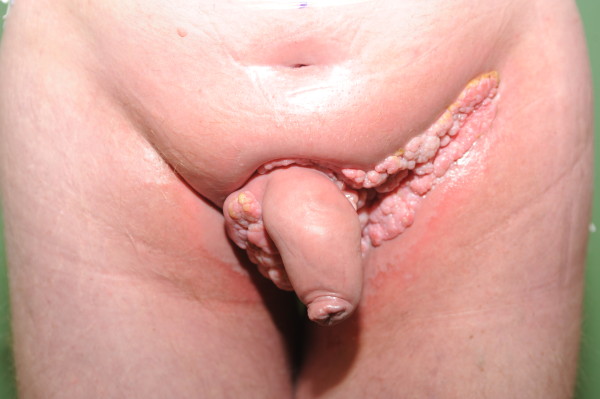
Tumor after therapy.

A reactive hypoparathyroidism with low phosphate caused by paraneoplastic hypercalcemia was suspected. After a single dose of pamidronate (60mg), saline infusions (3000mL/day) and diuretics (60mg furosemide/day) serum calcium was normalized, and the patient was discharged after six days. In follow-up examinations, slightly elevated calcium levels without clinical significance were detected. Due to the size of the tumor, a combined neoadjuvant therapy which involved radiotherapy of the tumor area up to 45Gy (gray) with a single dose of 1.8Gy plus acitretin was conducted (50mg daily for 43 days). The intensity-modulated radiation therapy technique (IMRT technique) was used. During this therapy, the patient was readmitted with fatigue and elevated calcium levels (3.36mmol/L). His serum phosphate level was normal. Our patient was again treated with a single dose of pamidronate (60mg), infusions and diuretics and discharged three days later. The neoadjuvant treatment resulted in a significant regression of the tumor mass. Pain while sitting had gone and calcium levels remained within normal range. During follow-up examinations (Figure 3) no further episodes of hypercalcemia occured.

## Discussion

While paraneoplastic hypercalcemia is well-known for many malignant diseases, it is rarely described in patients with localized skin tumors. In a single center study with 412 patients with cutaneous squamous cell carcinoma (SCC), Nicolae *et al*. reported a low PTH-independent hypercalcemia prevalence of 1.21% [7]. Furthermore, two cases with hypercalcemia-hyperleukocytosis paraneoplastic syndrome in cutaneous squamous cell carcinoma were described [8].

As far as it is known, there is only one case report of paraneoplastic hypercalcemia in Buschke-Löweinstein-tumor [9]. Hernandez *et al*. described a patient with Buschke-Löwenstein-tumor and elevated PTH-related protein (PTH-rp). After surgical treatment, hypercalcemia was normalized and PTH-rp became undetectable. In this patient, surgery finally resolved the problem. In our patient however, primary surgery was not an option due to the size and mass of the tumor. In the case of paraneoplastic syndrome some authors suggest a three point management plan: involving treatment of the primary tumor to eliminate the source of antigens; steroid based-immunosuppression to avoid antigen-antibody reactions; and symptomatic medication with bisphosphonates, diuretics and infusions to lower elevated calcium levels [10]. In this case, the use of steroids was withheld to await the effect of radiotherapy.

## Conclusions

Paraneoplastic hypercalcemia may occur in patients with Buschke-Löwenstein tumor. For patients who are not suitable for surgery, established medical therapies like bisphosphonates may be useful in addition to diuretics and infusions.

## Consent

Written informed consent was obtained from the patient for publishing this case report and any accompanying pictures. A copy of the written informed consent is available for review by the Editor-in-Chief-of this journal.

## Competing interests

The authors declare that they have no competing interests.

## Authors’ contributions

TL wrote this case report. TL and FM interpreted the data relating to the oncological disease. PH performed the combination of radiation and therapy. ML, PH and MG contributed to the writing and revision of the manuscript. All authors read and approved the manuscript.
